# Supplementary material for: BAD inactivation exacerbates rheumatoid arthritis pathology by promoting survival of sublining macrophages
Source: eLife. 2020 Dec 3;9:e56309. doi: 10.7554/eLife.56309 (PMC7714394; doi:10.7554/eLife.56309)
Supplement: Supplementary file 1. — (A) Immunoblotting analysis of BAD. (B) Immunoblotting analysis of pBAD(S136) in joint extracts after removing nonspecific bands using Bad−/− joint extracts. (C) Immunoblotting analysis of Vimentin. (D) Immunoblotting analysis of CD45R. (E) Immunoblotting analysis of F4/80. (F) Immunoblotting analysis of CD3. (G) Immunoblotting analysis of cleaved Casp-3 in mouse embryonic fibroblast (MEF) cells treated with TNFα (5 ng/ml) plus cycloheximide (CHX, 10 μg/ml). (H) Immunoblotting analysis of pBAD(S99) in THP-1 cells treated with TNFα (5 ng/ml). (I) Immunoblotting analysis of CD3 in THP-1 and Jurkat cell extracts. (J) Immunoblotting analysis of Vimentin in THP-1 and Hela cell extracts. (K) Immunoblotting analysis of CD68 in THP-1 and Jurkat cell extracts. [file elife-56309-supp1.docx]

**
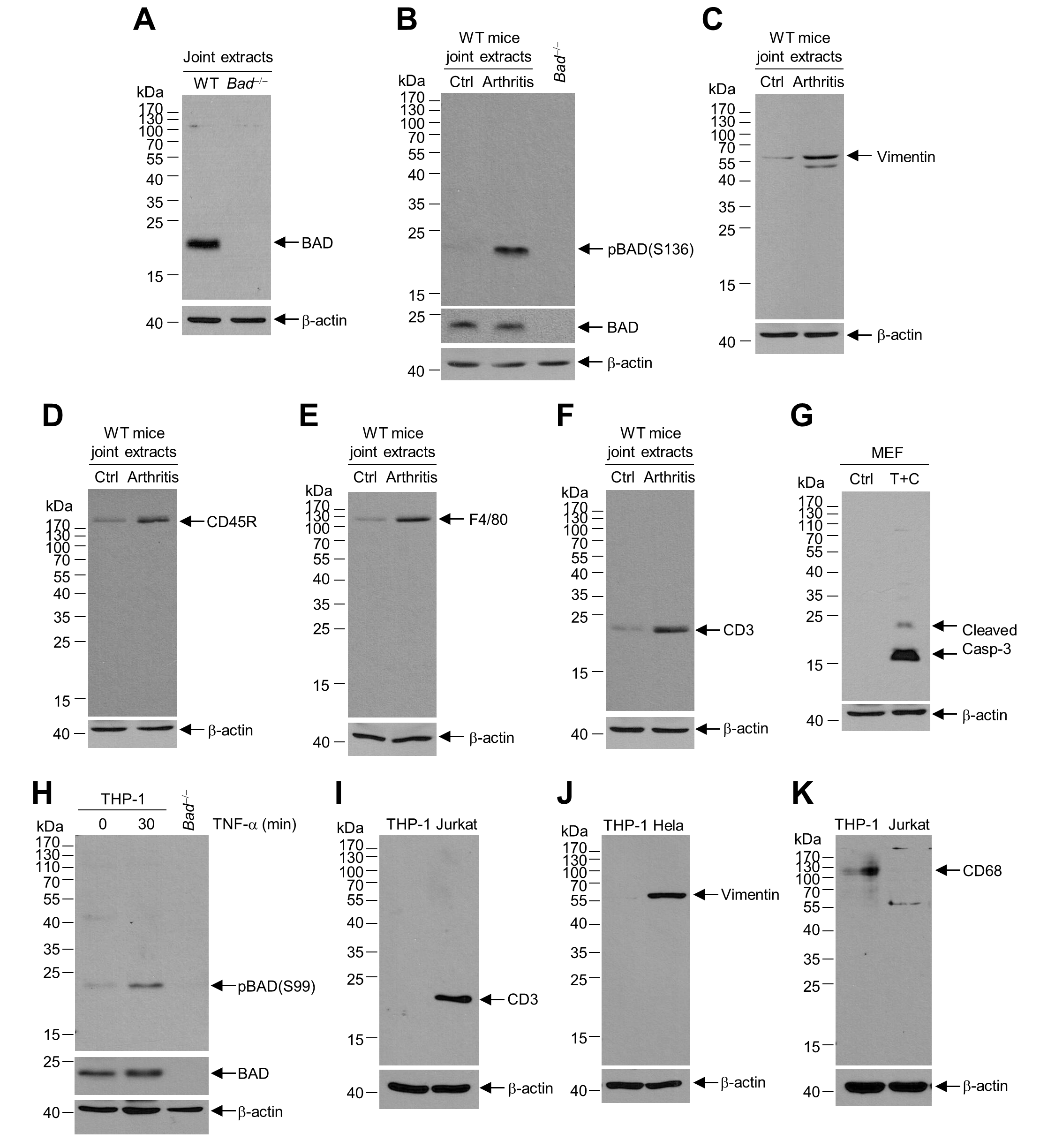
**

**Supplementary file 1.** Validation of antibodies used for immunofluorescence staining. **(A)** Immunoblotting analysis of BAD. **(B)** Immunoblotting analysis of pBAD(S136) in joint extracts after removing non-specific bands using *Bad*^−/−^ joint extracts. **(C)** Immunoblotting analysis of Vimentin. **(D)** Immunoblotting analysis of CD45R. **(E)** Immunoblotting analysis of F4/80. **(F)** Immunoblotting analysis of CD3. **(G)** Immunoblotting analysis of cleaved Casp-3 in mouse embryonic fibroblast (MEF) cells treated with TNFα (5 ng/ml) plus cycloheximide (CHX, 10 μg/ml). **(H)** Immunoblotting analysis of pBAD(S99) in THP-1 cells treated with TNFα (5 ng/ml). **(I)** Immunoblotting analysis of CD3 in THP-1 and Jurkat cell extracts. **(J)** Immunoblotting analysis of Vimentin in THP-1 and Hela cell extracts. **(K)** Immunoblotting analysis of CD68 in THP-1 and Jurkat cell extracts.
