# Supplementary material for: BAD inactivation exacerbates rheumatoid arthritis pathology by promoting survival of sublining macrophages
Source: eLife. 2020 Dec 3;9:e56309. doi: 10.7554/eLife.56309 (PMC7714394; doi:10.7554/eLife.56309)
Supplement: Supplementary file 2. [file elife-56309-supp2.docx]

**Original blots for Figure 2—figure supplement 4A:**

**
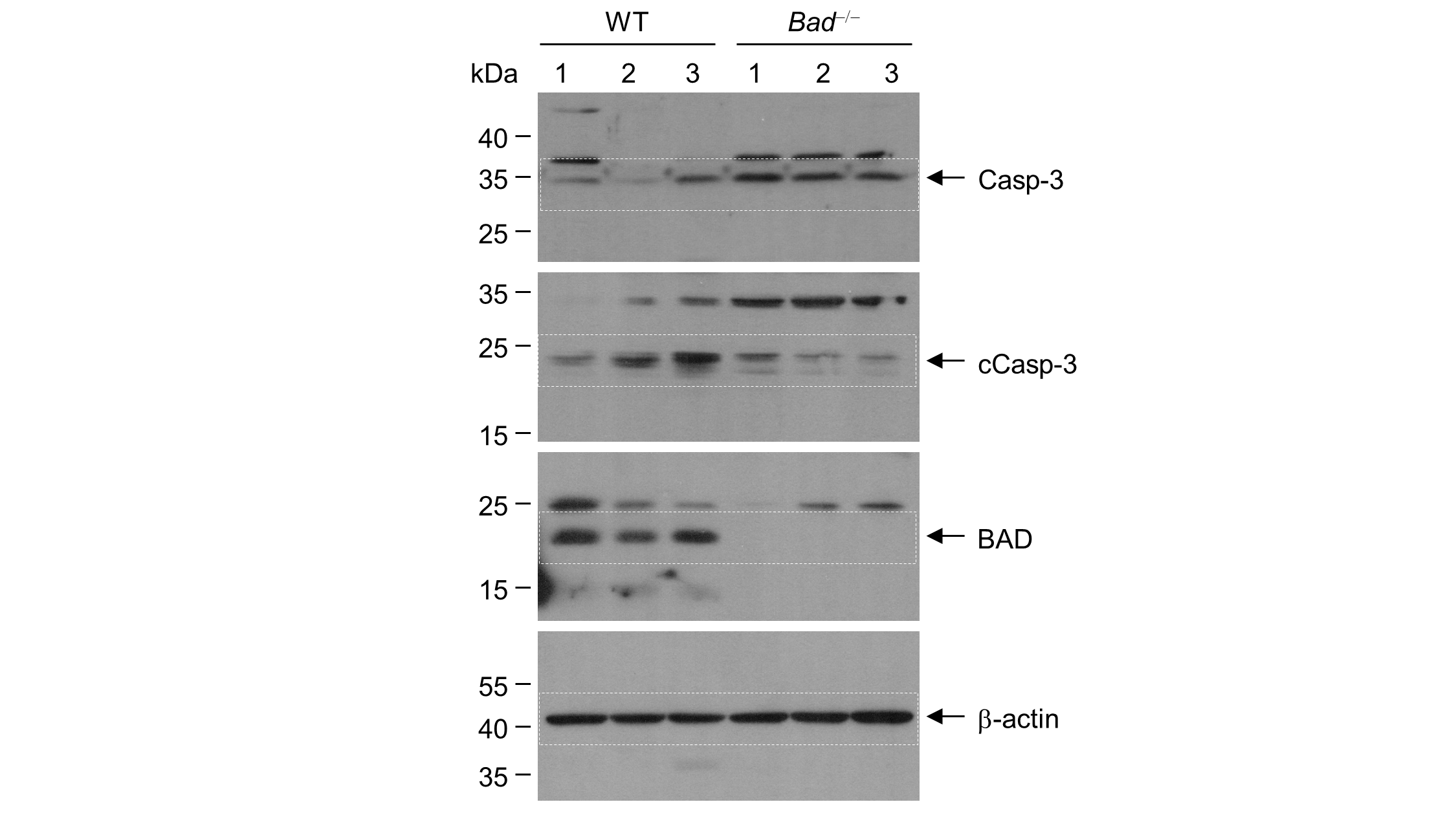
**

**Original blots for Figure 4A:**

**
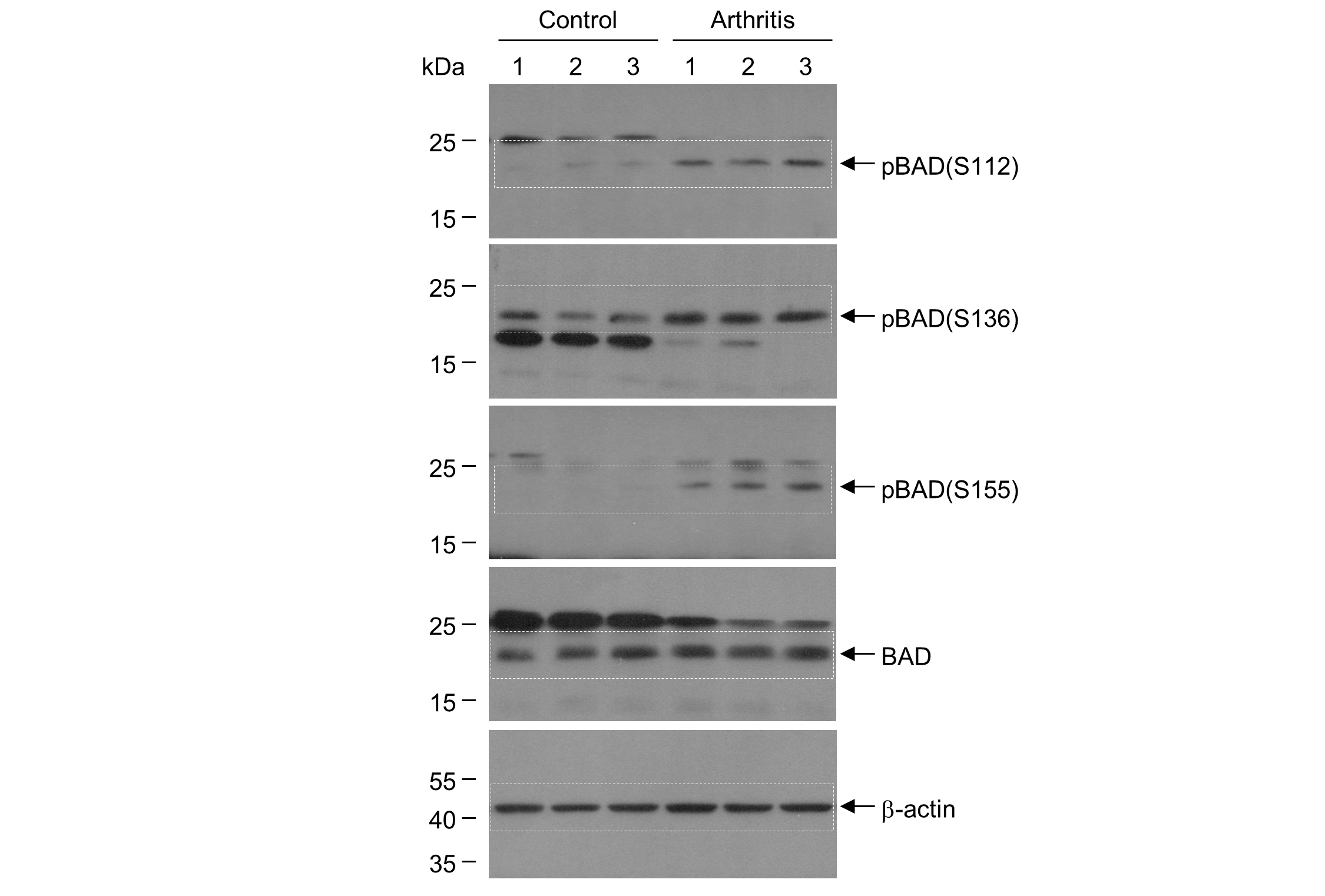
**

**Original blots for Figure 5E:**

**
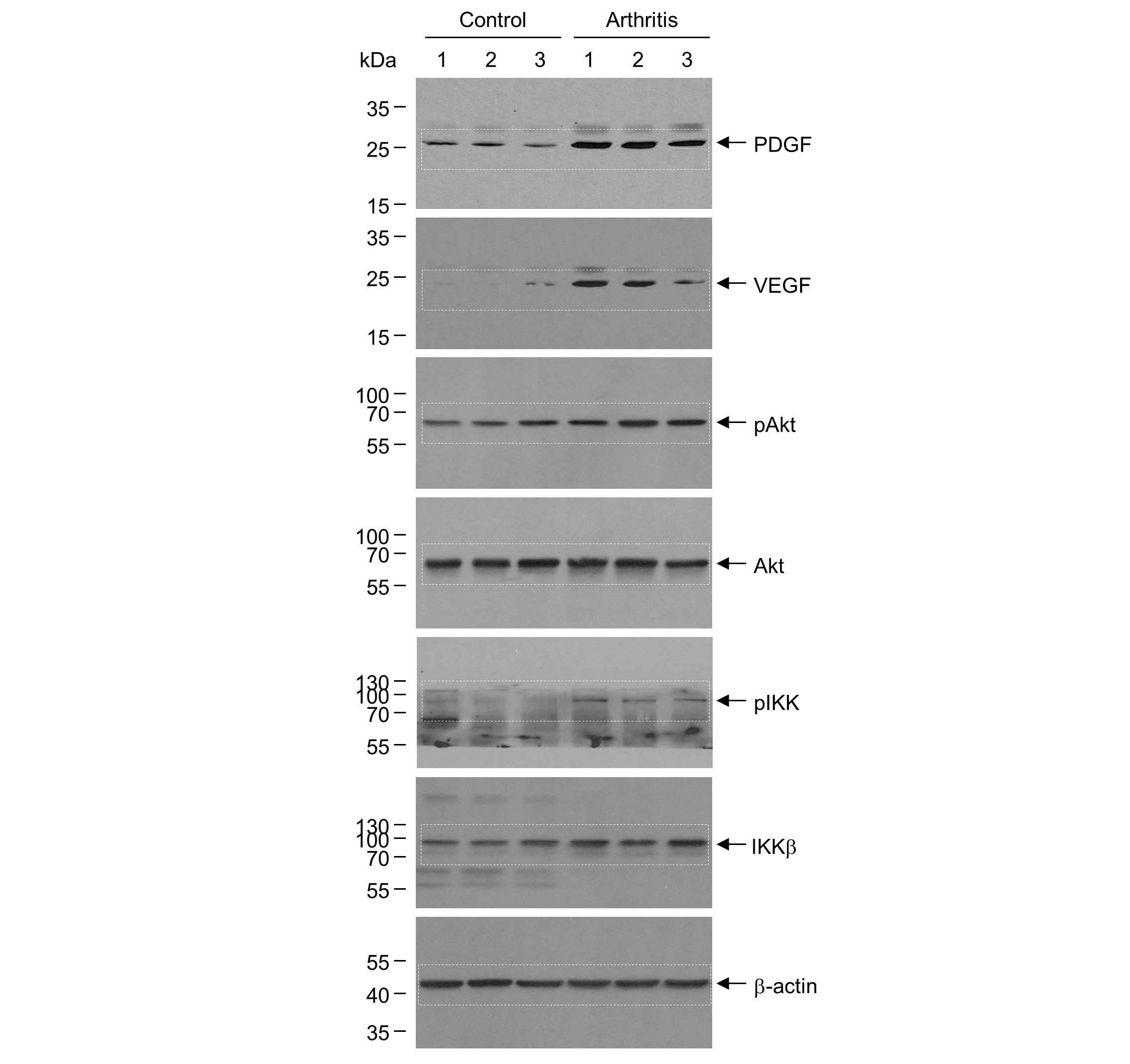
**

**Original blots for Figure 7A:**

**
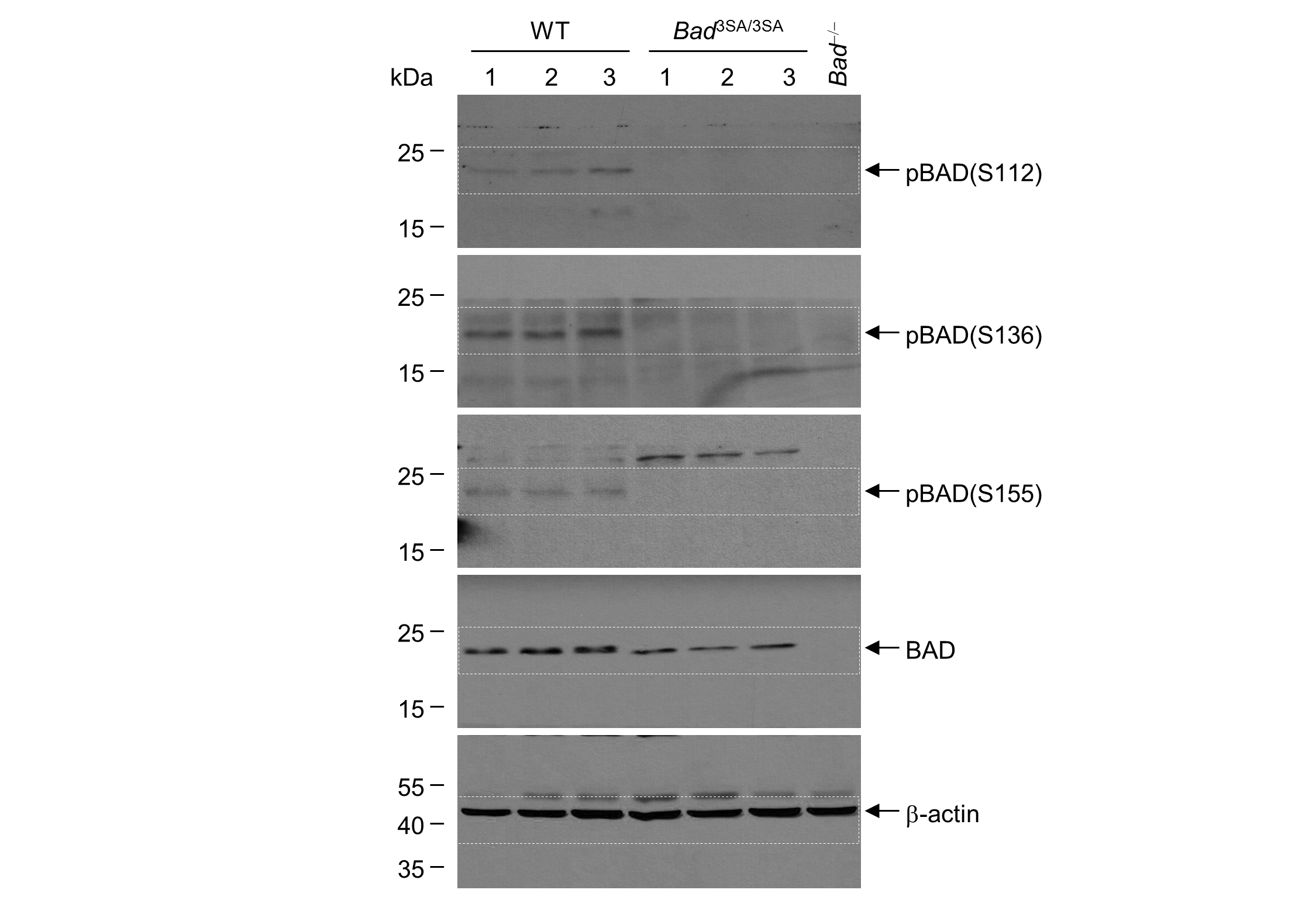
**

**Original blots for Figure 7G:**

**
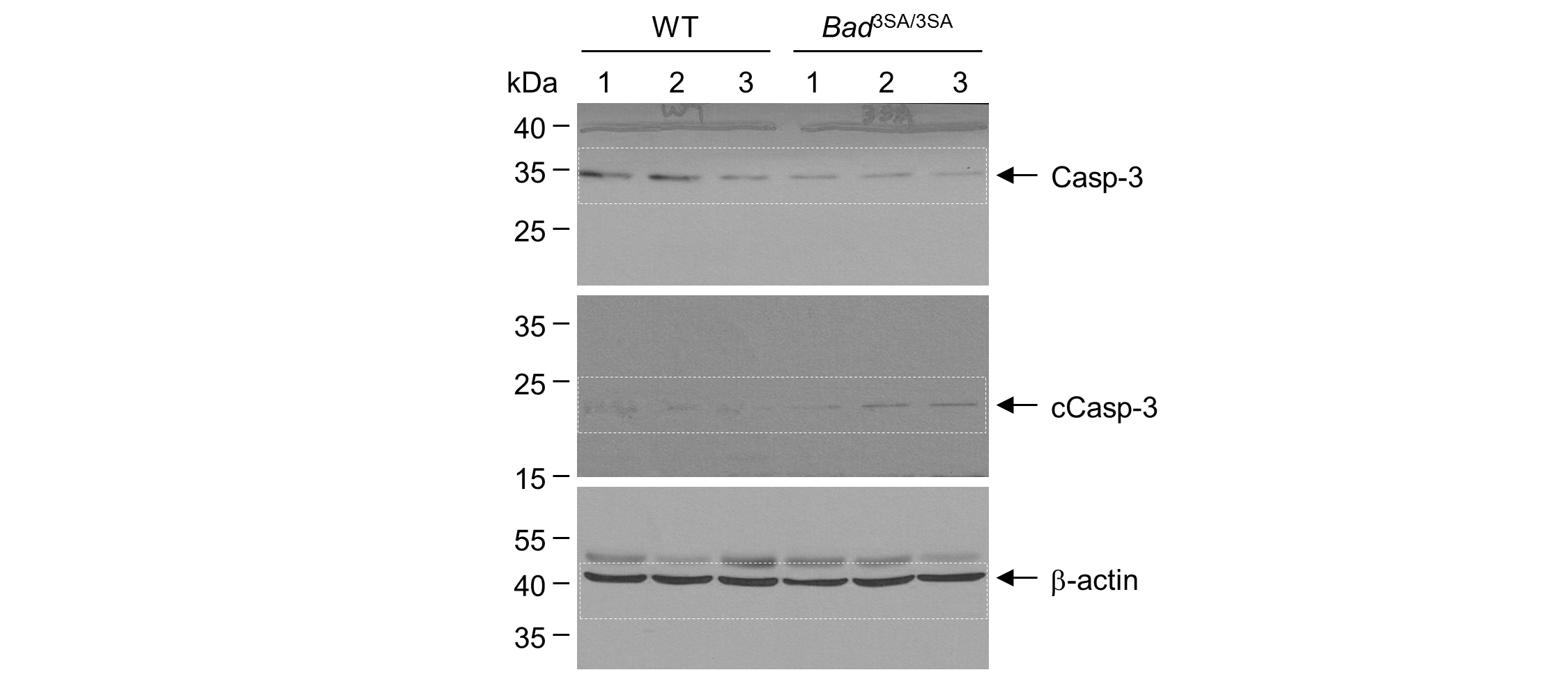
**

**Supplementary file 2.** Original blots for immunoblotting analysis in figures as indicated.
